# Non-specific interstitial pneumonia as the initial presentation of biphenotypic acute leukemia: a case report

**DOI:** 10.4076/1757-1626-2-8217

**Published:** 2009-08-04

**Authors:** Arun V Mohan, Venktesh R Ramnath, Eva Patalas, Eyal C Attar

**Affiliations:** 1 Department of Medicine, Cambridge Health Alliance1493 Cambridge Street, Cambridge, MA 02139USA; 2 Department of Pulmonary and Critical CareCambridge Health Alliance, 1493 Cambridge Street, Cambridge, MA 02139USA; 3 Department of Pathology, Cambridge Health Alliance1493 Cambridge Street, Cambridge, MA 02139USA; 4 Hematology/Oncology Unit, Massachusetts General Hospital32 Fruit Street, Yawkey 7A, Boston, MA 02114USA; 5 Harvard Medical School25 Shattuck Street, Boston, MA 02115USA

## Abstract

Nonspecific interstitial pneumonia has been linked to numerous etiologies including, most recently, haematologic malignancy. We present a 46-year-old woman with recent-onset rheumatologic illness who developed pulmonary symptoms as the presenting feature of biphenotypic acute leukaemia. Chest radiology demonstrated bilateral infiltrates, and lung biopsy revealed nonspecific interstitial pneumonia. Corticosteroid therapy resulted in resolution of both her pulmonary and rheumatologic symptoms, and her pulmonary symptoms did not recur following treatment of her leukemia. The case highlights the importance of searching for an underlying etiology when confronted with nonspecific interstitial pneumonia.

## Case presentation

A 46-year-old woman of Ethiopian descent presented to our emergency department with 3 weeks of worsening non-productive cough, dyspnea, fever, chills and night sweats. The patient’s medical history was significant for a three-year history of an uncharacterized rheumatologic illness marked by myalgias, arthralgias, two episodes of iridocyclitis, and a positive anti-nuclear antibody titer of 1:160, which had responded to treatment with systemic corticosteroids. Just three months prior to this presentation, she had developed thrombocytopenia with platelets to 37,000/mm^3^ which responded to prednisone (60 mg/day) which was slowly being tapered. Upon admission, her medications included prednisone (10 mg/day), calcium, and alendronate. Other past medical history included hypothyroidism and a completed treatment course for latent tuberculosis with isoniazid more than ten years prior. She worked as a cab driver and had a remote 20 pack-year history of smoking. She did not use alcohol or illicit drugs, and there was no family history of respiratory disease.

**Figure 1.**
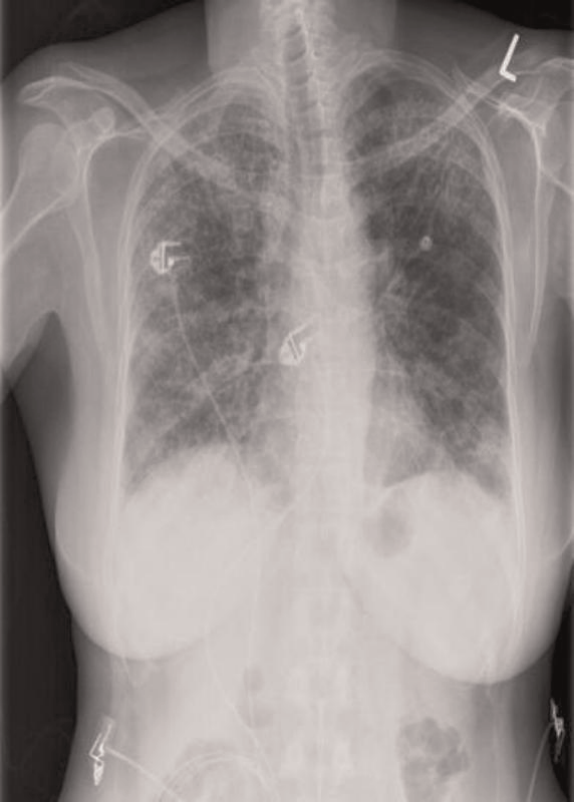
Chest radiograph showing diffuse, patchy bilateral interstitial infiltrates.

On examination, her temperature was 102.5 F with heart rate of 135 beats/minute, respirations of 33 breaths/min and oxygen saturation of 85% on 2 L/min supplemental oxygen. She was a moderately ill-appearing woman who had difficulty speaking in full sentences. Inspiratory crackles were present in bilateral lung fields and clubbing was absent. Her chest X-ray revealed diffusely increased interstitial prominence (Figure 1A). Laboratory evaluation indicated WBC of 6,700/mm^3^ with 16% neutrophils, 38% lymphocytes, and 38% atypical lymphocytes. All microbiologic studies were negative and bronchoalveolar lavage was non-diagnostic.

**Figure 2.**
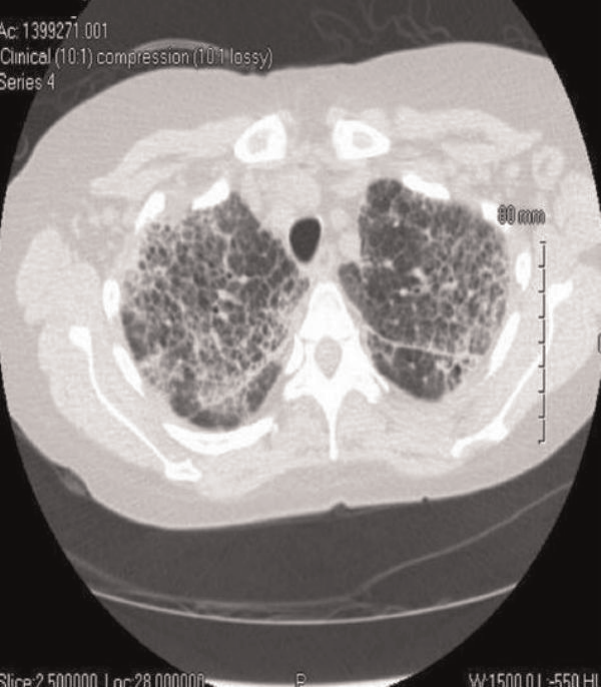
Chest CT revealing diffuse interstitial lung disease with septal interstitial thickening.

**Figure 3.**
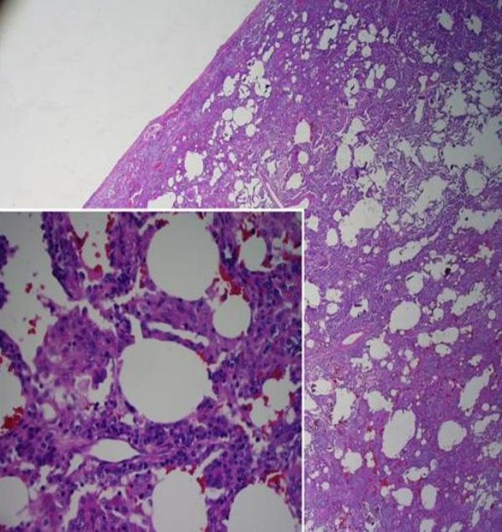
Low-power magnification (hematoxylin-eosin, original × 50) of lung biopsy showing expansion of the interstitium by inflammation and fibrosis. Inset: High-power magnification (hematoxylin-eosin, original × 400) demonstrating diffuse cellular interstitial pneumonitis with prominent lymphoplasmacytic infiltrates and intra-alveolar filling by histiocytes.

Chest CT showed diffuse patchy pulmonary interstitial opacities with peripheral predilection showing areas of septal thickening, ground glass, and subpleural honeycomb change (Figure 2) with para-aortic adenopathy. An open lung biopsy of the lingula revealed expansion of the interstitium by a diffuse lymphoplasmacytic infiltrate, intra-alveolar histiocytes and moderate interstitial fibrosis without fibroblastic foci or honeycomb lung (Figure 3). Immunostains confirmed a polymorphic population of T and B-lymphocytes with variable degrees of alveolar filling by benign macrophages with no evidence of leukemic infiltrates. This suggested a histopathologic diagnosis of nonspecific interstitial pneumonia.

Because of the patient’s lymphoplasmacytic lung infiltrate, peripheral blood atypical lymphocytes, and cytopenias, a bone marrow aspiration and biopsy was conducted to assess for the possibility of a hematologic malignancy. Her aspirate revealed 55% blasts with the following immunophenotype by flow cytometry: cytoplasmic CD3+, surface CD3-, CD7+, CD5+, CD2-, CD1-, CD33+, CD13-, CD117-, MPO-, CD79a-, CD56-, CD34+, terminal deoxytransferase (TdT)+, CD10+, CD45 dimly +, consistent with biphenotypic acute leukemia.

Given our initial suspicion for respiratory infection in the setting of chronic immunosuppression, we began treatment with moxifloxacin to cover community acquired bacterial pneumonia and trimethoprim-sulfamethoxazole with high-dose prednisone for the possibility of PCP. By the third day of hospitalization, however, the patient showed marked improvement in her respiratory symptoms without any evidence of infectious etiology and all antibiotics were withdrawn.

The patient’s respiratory function continued to improve over a period of five days; supplemental oxygen was discontinued and steroids were tapered. Two weeks after her presentation she received standard induction chemotherapy for AML consisting of daunorubicin 60 mg/m^2^ by intravenous push for three days and cytarabine 200 mg/m^2^/day by continuous infusion for seven days. Her day 14 bone marrow biopsy revealed an ablated marrow and complete remission was achieved on day 28. She subsequently underwent matched related donor allogeneic peripheral blood stem cell transplantation using reduced-intensity conditioning. There were no respiratory difficulties during the induction period or at any time following the transplantation. However, her disease relapsed 157 days after transplant and she passed away on day 205.

## Discussion

The lung pathology confirmed a diagnosis of nonspecific interstitial pneumonia. Nonspecific interstitial pneumonia (NSIP) represents a subtype of idiopathic interstitial pneumonia (IIP) with histologic features distinct from other IIPs, including varying degrees of cellular interstitial infiltrate (usually of mononuclear inflammatory cells) and interstitial fibrosis. NSIP may be seen in patients with a history of connective tissue diseases, toxic environmental exposures, drugs, hypersensitivity pneumonitis, infection, and prior lung injury [1]. Most recently, it has been reported in association with malignancy including leukemia [2,3]. While the pathogenesis of NSIP is unclear, both epithelial injury and immune activation have been implicated [4].

Clinically, patients with NSIP experience slowly progressive dyspnea in addition to other more common symptoms of chronic nonproductive cough, fatigue, malaise, anorexia and weight loss, all of which can be seen in chronic interstitial pulmonary disorders. Physical exam findings include the presence clubbing in approximately 10% of patients and bibasilar crackles in virtually all of them [1]. Laboratory findings are generally nonspecific but can frequently include an elevated erythrocyte sedimentation rate and lactate dehydrogenase level. Like other interstitial disorders, pulmonary function testing generally shows a decrease in diffusion to carbon monoxide and restrictive ventilatory deficit as marked by reductions in total lung capacity, residual volume, and functional residual capacity.

Chest radiographic findings are frequently nonspecific but can reveal increased interstitial markings with a basilar predominance; often high resolution computed tomography can be helpful. Bilateral, symmetric ground glass opacification is seen in about half of cases [5] which may indicate areas of cellular NSIP that have potential therapeutic and prognostic implications; presence of honeycombing and/or other fibrotic structural aberrations particularly in lower lung zones may indicate more fibrotic NSIP that may clinically approximate usual interstitial pneumonitis (UIP) more closely. Thus, despite suggestive imaging findings, surgical lung biopsy is often necessary to differentiate NSIP from other interstitial pulmonary pneumonitides, particularly UIP.

Although the response of IIPs to treatment is variable, NSIP generally carries a favorable prognosis, particularly compared to UIP [5]. The initial approach is to treat the underlying cause, including withdrawal of possible inciting medications or treatment of possible underlying connective tissue disorders. For patients in whom an underlying etiology cannot be found or who do not respond to this initial approach, corticosteroids are the mainstay of therapy. Immunosupressive drugs such as azathioprine and cyclophosphamide are an additional option for patients with more severe disease or who have an inadequate response to corticosteroids. According to a recent review, 10-year survival for patients with NSIP is 73.2% [5] and as many as 87% [1] of patients improve or recover completely.

In our patient, NSIP was likely a paraneoplastic phenomenon associated with the patient’s underlying biphenoptypic acute leukemia (BAL). BAL is a rare disease accounting for 3-10% of all acute leukemia [6]. Unlike myeloid or lymphoid-specific leukemias, BAL cells simultaneously coexpress both myeloid and lymphoid antigens. There is little evidence to guide the therapy of BAL and regimens for acute myeloblastic leukemia and acute lymphoblastic leukemia have been used [7]. In this case, although she had bone marrow and circulating blasts, her lung infiltrates were comprised of lymphoplasmacytes and polytypic lymphocytes rather than large cells consistent with myelolymphoblasts, suggesting a paraneoplastic process rather than simple leukemic cell infiltration.

Paraneoplastic autoimmune phenomena that include pulmonary infiltrates have been associated with a variety of hematologic disorders including myelodysplastic syndrome, chronic myelomonocytic leukemia, chronic lymphocytic leukemia, and lymphoma [8,9]. Although pulmonary infiltrates have not been reported in association with BAL prior to this case, autoimmune phenomena including pyoderma gangrenosum and osteoarthralgia have been associated with BAL [10].

In this patient, bronchoalveolar lavage and microbiologic studies did not disclose other causes for her NSIP. In addition, her symptoms promptly resolved with corticosteroid treatment and did not recur following treatment for leukemia. Although these findings do not definitively support a causative role of BAL, they are consistent with the notion that NSIP may represent a paraneoplastic process in some patients with underlying myelo/lymphoproliferative disorders.

## Abbreviations

BAL: Biphenotypic acute leukaemia
IIP: Idiopathic interstitial pneumonia
NSIP: Nonspecific interstitial pneumonia
UIP: Usual interstitial pneumonitis
WBC: White blood cells
